# Dynamic Mechanical Damage and Non-Shock initiation of a New Polymer Bonded Explosive during Penetration

**DOI:** 10.3390/polym12061342

**Published:** 2020-06-13

**Authors:** Xiao Li, Yizhi Liu, Yi Sun

**Affiliations:** Department of Astronautic Science and Mechanics, Harbin Institute of Technology, Harbin 150001, China; xli_90@163.com (X.L.); liuyizhi@hit.edu.cn (Y.L.)

**Keywords:** polymer-bonded explosives, mechanical damage, non-shock initiation, microcrack, frictional heating

## Abstract

Complexities of heating mechanisms make it difficult to investigate the safety of a polymer bonded explosive (PBX) charge of earth-penetrating-weapons (EPWs) during penetration. In this paper, the dynamic damage and non-shock initiation of PBX1314 (60 wt % hexahydro-1, 3, 5-trinitro-1, 3, 5-s-triazine (RDX), 16 wt % aluminum, 24 wt % hydroxy-terminated polybutadiene (HTPB)) during penetration is investigated through experiments and simulations. In the experiments, steel projectiles filled with PBX1314 are launched to penetrate concrete targets. In the results, non-shock initiations occur on the tail surface of PBX1314 along with mechanical damage of the tail and middle part of PBX1314. A dynamic damage and initiation model is proposed to characterize the effects of microcracks on the mechanical and thermal responses of PBX1314. Investigation based on the model suggests that microcrack interfacial friction plays significant roles in damage, heat generation and localization in PBX1314. A non-shock initiation criterion is developed based on macroscale variables in PBX1314. Numerical simulations of the penetration experiments are performed by using the proposed model and criterion. The mechanical damage and non-shock initiation of PBX1314 in the experiments are successfully predicted. The simulation results indicate that the tail of PBX1314 impacts the projectile repeatedly during penetration. Finally, the initiation criterion is satisfied because of frictional heat localization near microcrack surfaces and initiation is activated in the tail of PBX1314.

## 1. Introduction

Polymer-bonded explosive (PBX) is a kind of energetic material which consists of energetic grains, binder, and other additives, such as a desensitizing agent. A violent chemical reaction would occur when PBX is exposed to high-amplitude shock loadings. This is called shock detonation transition (SDT). Many theories and models [1,2,3] have been proposed to reveal the internal mechanisms of SDT. However, mild stimuli might also result in a reaction in PBX. The reaction is as violent as the reaction in SDT. This is called non-shock initiation [4]. Non-shock initiation has been of great concern of researchers for several decades but the study is still at an exploratory stage [5]. It is important for the safety assessment of military weapons. Earth-penetrating-weapons (EPWs) are designed to penetrate defensive structures and then destroy underground targets. PBX in the EPWs would experience complex loading process during penetration. Mechanical damage might occur near initial material flaws in the PBX. The coupling of mechanical, thermal, and chemical effects in the damaged regions would lead to a high temperature in local sites in the PBX. The sites are named as hotspots. The formation and evolution of hotspots might lead to initiation, which poses serious threats to the safety of the PBX. Once non-shock initiation occurs in the PBX during penetration, the EPWs would explode before reaching the intended penetration depth and the underground targets could not be effectively destroyed. Therefore, it is urgent to investigate the mechanisms of non-shock initiation of the PBX and provide reliable methods to safety assessment of the EPWs during penetration. The non-shock initiation has a close relationship with PBX compositions, manufacturing techniques, and loads. During initiation, mechanical energy is converted to heat in the material and localizes in mesoscale sites. The heat localization leads to the formation of hotspots which are potential initiation locations.

A series of mechanisms have been proposed to account for the energy conversion in non-shock initiation [6]. Barua et al. [7,8] develops the cohesive finite element method (CFEM) to numerically investigate the formation of microcracks, frictional heating, and energy localization in PBX9501 (95 wt % octahydro-1,3,5,7-tetranitro-1,2,3,5-tetrazocine (HMX), 5 wt % binder). A criterion has been developed to distinguish whether the sets of hotspots are potential initiation sites [9]. In consideration of the microstructure, crystal physical property and binder viscoelasticity, investigations of hotspot formation in granular explosive and PBX indicate that heat localization is related to crystal anisotropy, microstructural heterogeneity [10,11,12], crystal sliding, and localized plastic deformation [13,14]. A phase field damage model is constructed to study the mechanical and thermal response of an HMX particle, which is embedded in the binder matrix [15,16]. The calculations reveal that the localized damage at the particle-binder interface contributes to the formation of hot spots. Based on the analysis of penny-shaped cracks, a statistical crack model (SCRAM) is presented [5,17] to describe the opening, shear, growth, coalescence of microcracks in brittle materials. Crack friction is assumed to be the heating mechanism. The calculated crack damage and non-shock initiation of PBX9501 agree well with experiments [5,17,18]. Visco-SCRAM [4] and VEP-DCA [19] were developed by other researchers. They both adopt the crack friction heating mechanism. The mechanical and thermal responses of PBX9501 under impact loadings have been researched [4,19,20,21]. The calculations [4,5,19] give evidence to the crack friction heating assumption and good prediction for experiments. 

The above theoretical models give insights into the damage and initiation mechanisms of PBX under impact loadings. The corresponding calculations reasonably predict the mechanical, thermal and chemical responses of PBX in experiments. However, only simple impact loads, for example uniaxial compression at constant strain rates [2,3], are considered in the above research. They are very different from the complex loads that PBX structure would endure in engineering. Moreover, the above investigations focus on the pressed energetic material PBX9501. Studies on the damage and non-shock initiation of cast energetic material, which is widely used in military weapons, is rarely reported.

PBX1314 is a cast energetic material with 60 wt % hexahydro-1,3,5-trinitro-1,3,5-s-triazine (RDX), 16 wt % aluminum powder and 24 wt % hydroxy-terminated polybutadiene (HTPB). The binder makes PBX1314 soft under uniaxial compression [22], uniaxial tension [23], multi-axial compression [24] and gives it low sensitivity under shock [25]. In impact damage experiments [26], microcracks nucleation and growth are observed. The simulations based on the Visco-SCRAM provides the evidence that micorcracks have an effect on determining the mechanical response of PBX1314 [26]. In engineering applications, impact loads to PBX1314 main explosive sustain thousands of microseconds and alternate between compression and tension repeatedly when projectiles penetrate defensive structures. The effects of microcracks and binder on mechanical damage and the non-shock initiation of PBX1314 under such loadings remains to be researched.

In this paper, a systematic investigation on the mechanical damage and non-shock initiation of cast PBX1314 in projectiles during penetration is conducted. The damage and non-shock initiation of PBX1314 in penetration are studied by examining the structural integrity and scorch of the PBX1314 after experiments. The mechanical, thermal, and chemical responses of PBX1314 during penetration are numerically simulated. A dynamic damage model based on microcracks is presented. A non-shock initiation criterion is proposed based on macroscale variables. The results are generally in accordance with the experiment.

## 2. Materials and Experiments 

### 2.1. PBX1314 Composition

The cast PBX1314 consists of 60 wt % hexahydro-1,3,5-trinitro-1,3,5-s-triazine (RDX) particles, 16 wt % aluminum, and 24 wt % hydroxyterminated polybutadieneopening (HTPB) binder. The PBX1314 was bought from Hubei Institute of Aerospace Chemical Technology (Wuhan, China). The mass density of PBX1314 is 1.65 g/cm^3^. The size of RDX particles ranges from 50 μm to 200 μm. The binder content of PBX1314 is higher than those of widely investigated explosives such as PBX9501 (95% HMX, 3% BDNPA/F, and 3% estane) and LX-04 (85% HMX and 15% Viton) [27]. The binder provides a cushion for energetic grains when the material is exposed to exterior loads. In the case of initiation and detonation, the exothermic reaction of aluminum could produce an increased blast. The scanning electron microscope (SEM) micrograph of PBX1314 is given by Xiao [22].

### 2.2. Penetration Experiment

**Figure 1:**
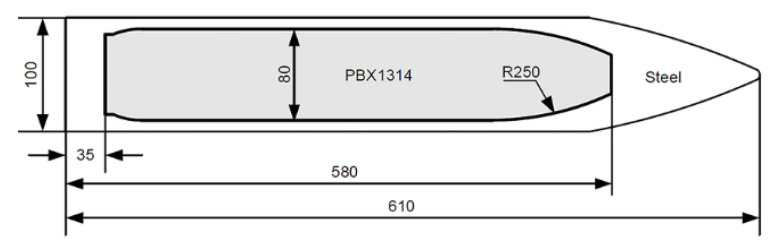
Geometries of the projectile and the inner PBX1314 main explosive. All the size parameters are in millimeters.

The geometries of the steel projectile and PBX1314 are illustrated in Figure 1. The density of the steel was 8020 kg/m^3^, the elastic modulus was 200 GPa, and the yielding strength was 1.72 GPa. A polyurethane liner is sprayed on the inner surface of the projectile. Then PBX1314 is cast into the steel projectile. The concrete is mixed by sands and cements. The mass density of the concrete is 2420 kg/m^3^ and the modulus is 34 GPa. The unconfined compressive strength is 45 MPa and the tensile strength is 4 MPa. The concrete targets are cylinders with 2 m diameter and 1.8 m height. The projectiles are launched by a 105-mm smooth-bore powder gun perpendicular to the front of the targets. The flying velocities of the projectiles are measured by a high-speed photography system. 

**Figure 2:**
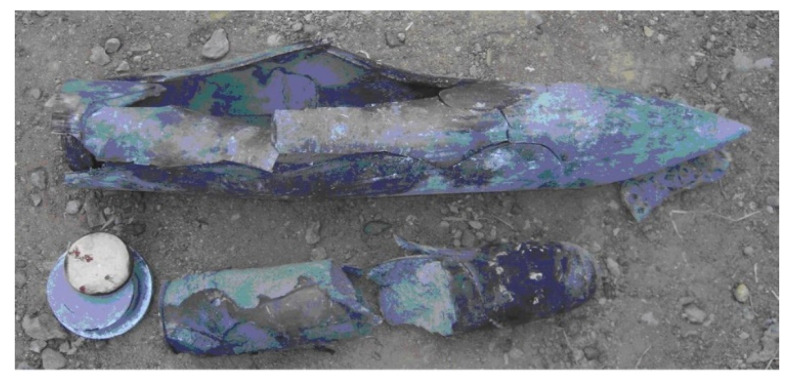
Penetration experiment results.

The projectile and PBX1314 after a 602 m/s penetration experiment is displayed in Figure 2. The head of the projectile deformed slightly. Only a little mass abrasion occurred. However, the cylindrical part of the projectile expanded and fractured. It resulted from the non-shock initiation of PBX1314. The PBX1314 broke in two when it was taken out of the projectile. The mechanically damaged zone was distributed from the tail to the middle part of PBX1314. Initiation occurred in the tail part in a certain moment during penetration. The generated gas products and liberated energy during the initiation raised the internal pressure and temperature of the projectile. The increase in temperature and internal pressure led to the expansion and fracture of the projectile. In addition, the experimental results indicate that the initiation finally quenched rather than growing to detonation. 

## 3. Dynamic Damage Model of PBX1314

Damage and fracture of PBX1314 in the penetration experiments may results from the nucleation and growth of microcracks. A dynamic damage model is developed to characterize the dynamic mechanical properties of PBX1314. The effect of microcracks on stress–strain responses and damage evolution is studied.

### 3.1. Dynamic Damage Model

Take into consideration of the microcracks and viscoelastic binder, the dynamic damage model of PBX1314 is built by the combination of a microcrack model [28] and a general *N*-component Maxwell model. The decomposition of the total strain *ε_ij_* of PBX1314 in rate form is

(1) $${\dot{\varepsilon }}_{ij}={\dot{\varepsilon }}_{m}\delta_{ij}+{\dot{e}}_{ij}$$

where *ε_m_* and *e_ij_* are hydrostatic strain and deviatoric strain, respectively. *δ_ij_* is the Kronecker symbol. The dots denote the time rate of the corresponding variables. The deviatoric strain rate consists of two parts

(2) $${\dot{e}}_{ij}={\dot{e}}_{ij}^{ve}+{\dot{e}}_{ij}^{c}$$

The superscript *ve* and *c* represent the effects of viscoelastic binder and microcracks. 

The decomposition of the total stress *σ_ij_* of PBX1314 in rate form is

(3) $${\dot{\sigma }}_{ij}={\dot{\sigma }}_{m}\delta_{ij}+{\dot{s}}_{ij}$$

in which *σ_m_* and *s_ij_* are pressure and deviatoric stress, respectively. The constitutive law of PBX1314 in hydrostatic space is [4]

(4) $${\dot{\sigma }}_{ij}=3K{\dot{\varepsilon }}_{m}$$

where *K* is the bulk modulus of PBX1314. 

In deviatoric space, the general Maxwell model which is in series with the microcrack model. The constitutive law of the general Maxwell model is first analyzed. In a single-element Maxwell model, an elastic element is in series with a viscous element. The constitutive laws of the elastic element and viscous element are

(5) $$s_{ij}^{e}=2Ge_{ij}^{e}$$

(6) $$s_{ij}^{v}=2\eta {\dot{e}}_{ij}^{v}$$

where *G* is the shear modulus of the elastic element and *η* is the viscous coefficient of the viscous element. The derivative of Equation (5) with respect to time is

(7) $${\dot{s}}_{ij}^{e}=2G{\dot{e}}_{ij}^{e}$$

As the elastic element is in series with the viscous element in the Maxwell model, the deviatoric stresses of the two elements are the same as the deviatoric stress of the Maxwell model, while the addition of the deviatoric strains of the two elements equals the deviatoric strain of the Maxwell model

(8) $$s_{ij}^{ve}=s_{ij}^{e}=s_{ij}^{v}$$

(9) $$e_{ij}^{ve}=e_{ij}^{e}+e_{ij}^{v}$$

The derivative of Equation (9) with respect to time is

(10) $${\dot{e}}_{ij}^{ve}={\dot{e}}_{ij}^{e}+{\dot{e}}_{ij}^{v}$$

Combining Equation (6), Equation (7), Equation (8), and Equation (10), together, the constitutive law of the Maxwell model could be obtained

(11) $${\dot{s}}_{ij}^{ve}=2G{\dot{e}}_{ij}^{ve}-s_{ij}^{ve}/\tau$$

where *τ* = *η*/*G* is the relaxation time. In an *N*-component Maxwell model, all the components have the same deviatoric strain and it equals the global deviatoric strain of the *N*-component Maxwell model. The total deviatoric stress of the *N*-component Maxwell model equals the superposition of the deviatoric stresses of all the components. That is

(12) $${\dot{s}}_{ij}=\sum_{n=1}^{N}{\dot{s}}_{ij}^{ve\left(n\right)}$$

According to Equation (11), the deviatoric constitutive law of each component is

(13) $${\dot{s}}_{ij}^{ve\left(n\right)}=2G^{\left(n\right)}{\dot{e}}_{ij}^{ve}-s_{ij}^{ve\left(n\right)}/\tau^{\left(n\right)}$$

where *G*^(*n*)^ and *τ*^(*n*)^ are the shear modulus and relaxation time of the *n*-th component in the general Maxwell model. The deviatoric constitutive law of the *N*-component Maxwell model is obtained by Equations (12) and (13)

(14) $${\dot{s}}_{ij}=\sum_{n=1}^{N}\left(2G^{\left(n\right)}{\dot{e}}_{ij}^{ve}-s^{\left(n\right)}{}_{ij}/\tau^{\left(n\right)}\right)$$

The effects of ensembles of microcracks on the mechanical properties of PBX1314 are characterized by the employment of the microcrack model [28]. 

In the present work, the feature size of the PBX1314 structures, *L*_s_, is in order of magnitude 1 to 10 cm [20,24], while the feature size of the microcracks, *L*_c_, is in the order of magnitude 1 to 10 μm. A representative element volume (RVE) is extracted from the PBX1314 structures. The feature size of the RVE, *L*_R_, should be much less than *L*_s_ so that the RVE is infinitesimal in macroscale and the stress and strain distribution in the RVE is homogeneous. Besides, *L*_R_ should be much larger than *L*_c_ so that the RVE contains a large number of microcracks and the RVE could characterize the statistical properties of the microcracks. The distribution of microcracks in the RVE are assumed to be isotropic and the interactions among different microcracks are ignored. In each direction, the radius distribution of the microcracks is assumed to be exponential. The additional strains of the RVE due to the existence of one microcrack under the condition of remote stress are obtained by Addessio [28]. The total deviatoric strain resulting from the ensembles of microcracks of the RVE is obtained by integrating the individual microcrack strains over a material volume, all crack sizes and all directions [28,29]. In this way, the mechanical properties of the RVE in mesoscale is converted into a macroscale constitutive law through statistical homogenization methods. The integration gives the deviatoric constitutive law [28]

(15) $$e_{ij}^{c}=\beta^{e}c_{a}^{3}s_{ij}$$

in which *β^e^* = 2(5 − *ν*)*β* for tensed cases and *β^e^* = 6*β* for compressed cases. The ν is the Poisson ratio of PBX1314. The parameter *β* is expressed as *β =* (64*π*/15)[(1 − *ν*)(2 − *ν*)](*N*_0_/*G*′). *N*_0_ is the initial microcracks number density, *c*_a_ is the size of an equivalent microcrack. In Equation (15), the mechanical properties of ensembles of microcracks are characterized by one equivalent microcrack for which the radius is c_a_. 

By multiplying 2*G*′, Equation (15) is transformed to be

(16) $$2G\prime \varepsilon_{ij}^{c}={\left(c_{a}/a^{\prime }\right)}^{3}s_{ij}$$

in which *a*’ = *a*(3/*κ*)^1/3^, *a* is the initial flaw size of PBX1314. For opened cracks *κ =* 5−ν, while for closed cracks *κ =* 3. The relation between *a*’ and *β*^e^ is

(17) $$a^{\prime }={\left(2G\prime \beta^{e}\right)}^{1/3}$$

The deviatoric constitutive law of PBX1314 is obtained by the combination of Equations (2), (14), (16) and expressed as

(18) $${\dot{s}}_{ij}=\left[2G^{\prime }{\dot{e}}_{ij}-\sum_{1}^{N}\frac{s_{ij}^{\left(n\right)}}{\tau^{\left(n\right)}}-3{\left(\frac{c_{a}}{a^{\prime }}\right)}^{3}\frac{{\dot{c}}_{a}}{a^{\prime }}s_{ij}\right]/\left[1+{\left(\frac{c_{a}}{a^{\prime }}\right)}^{3}\right]$$

Equations (4) and (8) comprise the constitutive law of PBX1314. 

In fracture mechanics theories, cracks become unstable once energy release rate *g* exceeds a critical level. The *g* of a microcrack with radius *c*_a_ is [5]

(19) $$g=\left[4\left(1-\nu \right)f\left(\sigma ,n\right)c_{a}\right]/\left[\pi \left(2-\nu \right)G^{\prime }\right]$$

where *f* is a stress function which depends on the stress state and the normal of the microcrack. For a tensed crack

(20) $$f\left(\sigma ,n\right)=\left(1-\nu /2\right)\sigma_{n}^{2}+s_{n}^{2}$$

while for a compressed crack

(21) $$f\left(\sigma ,n\right)={\langle s_{n}+\mu_{s}\sigma_{n}\rangle }^{2}$$

where the Macaulay bracket <·> means that it takes the value of the inside formula if the inside formula is positive and takes zero if the inside formula is zero or negative. In a microelement of PBX1314, the microcracks are in the same stress state, while their mechanical responses varies with their own normal directions. For a certain stress state, there exists a critical direction in which the microcracks have the maximum energy release rate. These Microcracks are more unstable than others. A dominant crack [29,30] with radius *c*_a_ and locates in this direction is defined to statistically characterize the effects of all microcrracks. The stress function of the dominant microcrack is [30]

(22) $$f=\{\begin{array}{ll} \left(1-\frac{\nu }{2}\right)\sigma_{1}^{2}, & -\left(1-\nu \right)<r\leq 1\\ \left[\left(\frac{2}{\nu }-1\right){\left(1+r\right)}^{2}+{\left(1-r\right)}^{2}\right]\frac{\sigma_{1}^{2}}{4}, & -1<r\leq -\left(1-\nu \right)\\ \left[\left(\sqrt{\mu^{2}+1}-\mu \right)-\left(\sqrt{\mu^{2}+1}+\mu \right)\frac{1}{r}\right]\frac{\sigma_{3}^{2}}{4}, & \begin{array}{l} r\leq -{\left(\sqrt{\mu^{2}+1}+\mu \right)}^{2}\\ \mathrm{or}\;\;r>{\left(\sqrt{\mu^{2}+1}+\mu \right)}^{2} \end{array}\\ -\sigma_{1}\sigma_{3}, & -{\left(\sqrt{\mu^{2}+1}+\mu \right)}^{2}<r\leq -1\\ 0, & 1<r\leq {\left(\sqrt{\mu^{2}+1}+\mu \right)}^{2} \end{array}$$

where *r* is the ratio of the third principle stress to the first principle stress. The five states in Equation (22) mean pure open, open with shear, pure shear, close with shear, and friction locked of the dominant crack, respectively. The dominant crack growth rate is [5]

(23) $${\dot{c}}_{a}=\{\begin{array}{ll} v_{\max}{\left(g/g_{1}\right)}^{m}, & g\leq g_{tr}\\ v_{\max}\left(1-g_{c}/g\right), & g>g_{tr} \end{array}$$

in which *g*_tr_ is a transition threshold, *g*_c_ is twice the effective surface energy γ_0_, *v*_max_ is the Rayleigh wave speed, *m* is a coefficient. According to the continuity, *g*_tr_ and *g*_1_ are obtained

(24) $$g_{tr}=\left(1+\frac{1}{m}\right)g_{c}$$

(25) $$g_{1}={\left(m+1\right)}^{1/m}\left(1+\frac{1}{m}\right)g_{c}$$

The growth of microcracks results in damage in PBX1314. The damage is defined as

(26) $$D=\frac{c_{a}^{3}}{c_{a}^{3}+a^{3}}$$

### 3.2. Effects of the Microcracks and Binder on Dynamic Mechanical Properties of PBX1314

The dynamic damage model is converted into a visual user material subroutine (VUMAT) code by using FORTRAN. The VUMAT code is implemented into ABAQUS. The ability of the model in characterizing dynamic mechanical responses of PBX1314 at different stress states and strain rates are examined. The effects of microcracks and binder on the dynamic mechanical properties of PBX1314 are studied. The responses of PBX9501 under similar loads are calculated for comparison. The mechanical parameters of PBX1314 are listed in Table 1 (in the third column of Table 1, the parameters from *ρ* to *E* are related to non-shock initiation of PBX1314. They will be introduced and used in Section 4. As the parameters related to the dynamic damage model and those related to the initiation model will be together employed in Section 5, we list the two categories of parameters together in Table 1.) and parameters of PBX9501 are given by Yang [19]. 

**Figure 3:**
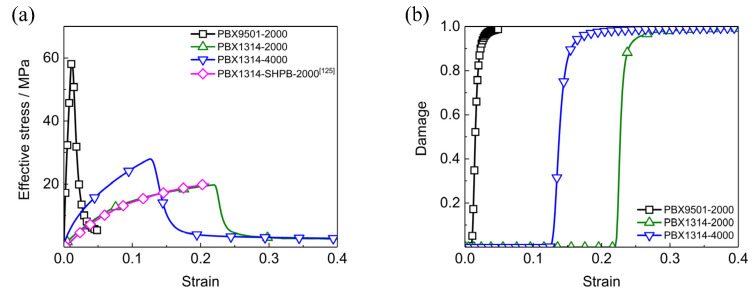
Dynamic mechanical responses of PBX1314 and PBX9501 under uniaxial compressions. (**a**) Stress–strain curves, (**b**) damage–strain curves.

The dynamic mechanical responses of PBX1314 under uniaxial compression at different strain rates are calculated and displayed in Figure 3. At the strain rate of 2000 s^−1^, the stress of PBX1314 is at a low level and PBX1314 exhibits viscoelastic properties in the beginning stage. As the strain exceeds 0.2, the increasing stress results in high energy release rates according to Equations (19) and (21). The continuously increasing energy release rates lead to fast crack growth when the strain reaches the critical value of 0.22. The fast crack growth causes stress softening and damage accumulation. The stress-strain curve of PBX1314 at 2000 s^−1^ strain rate in Figure 3. agrees well with the SHPB experiment data [22]. This demonstrates the ability of the dynamic damage model in predicting the dynamic compressive properties of PBX1314. In addition, the difference between the curves of 2000 and 4000 s^−1^ indicates the strain-rate dependence of the dynamic mechanic responses of PBX1314. This dependence is coincide with the experiment results [22]. 

In Figure 3a, PBX1314 is shown to have higher critical softening strains but lower stress compared to PBX9501. This is attributed to the high content binder by which the modulus of PBX1314 is weakened. Besides, it is the contact and compression among energetic particles that lead to microcracks growth and damage accumulation. Energetic particles distribute sparsely in PBX1314 due to the high content binder. PBX1314 undergoes higher compressive strain than PBX9501 before the particle contact and compression.

**Figure 4:**
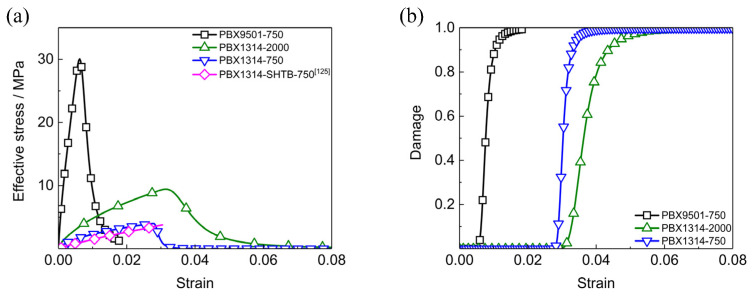
Dynamic mechanical responses of PBX1314 and PBX9501 under uniaxial tensions. (**a**) Stress–strain curves, (**b**) damage–strain curves.

Dynamic mechanical responses of PBX1314 under uniaxial tension at different strain rates are calculated and displayed in Figure 4. In Figure 4a, the stress–strain curve of PBX1314 at the strain rate of 750 s^−1^ shows the same tensile strength as the SHTB experiment data [23]. This verifies the ability of the dynamic damage model of characterizing the dynamic tensile properties of PBX1314. The effect of binder in reducing modulus and enlarge softening strain in PBX1314 could also be seen. The curves in Figure 4a have smaller dynamic strengths and stress softening strains than those in Figure 3a. This is due to the different responses of the dominant microcracks under tension and compression. The dominant microcracks close and shear when PBX1314 is compressed while purely open when it is tensed. The tensile load leads to higher energy release rates if the tensile and compressive loads are in the same magnitude. In another words, the stress level at which the high-speed crack growth is activated is much lower when PBX1314 is tensed.

**Figure 5:**
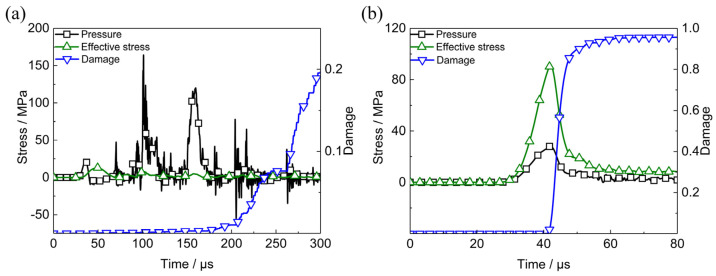
Dynamic mechanical responses of PBX1314 and PBX9501 under multi axial compression. (**a**) Pressure, effective stress and damage of PBX1314, (**b**) pressure, effective stress and damage of PBX9501.

The mechanical responses of PBX1314 in multi-axial impact experiments [26] in which the input bar is 60 mm in length and the impact velocity is 31.8 m/s are calculated and shown in Figure 5a. The first peak of pressure is 30 MPa, which agrees well with the experimental data 29.075 MPa [26]. In the beginning stage, the pressure is higher than the effective stress. All microcracks in the specimen are friction-locked and no crack growth occurs. After the initial impact, the stress state of the specimen undergoes a complex evolution process. Tensile hydrostatic stress and effective stress in corresponding periods result in microcracks growth and material damage. The curves in Figure 5a indicate the ability of the dynamic damage model in predicting the dynamic multi-axial mechanical responses of PBX1314. 

The dynamic mechanical responses of PBX9501 under the same multi-axial compression loading is calculated and shown in Figure 5b. The initial impact causes severe shear deformation in the specimen and the fast growth of microcracks is activated immediately. Comparison of the responses of PBX1314 and PBX9501 suggests that the binder has significant effects on the dynamic mechanical responses of PBX1314. 

## 4. Non-Shock Initiation Model of PBX1314

**Figure 6:**
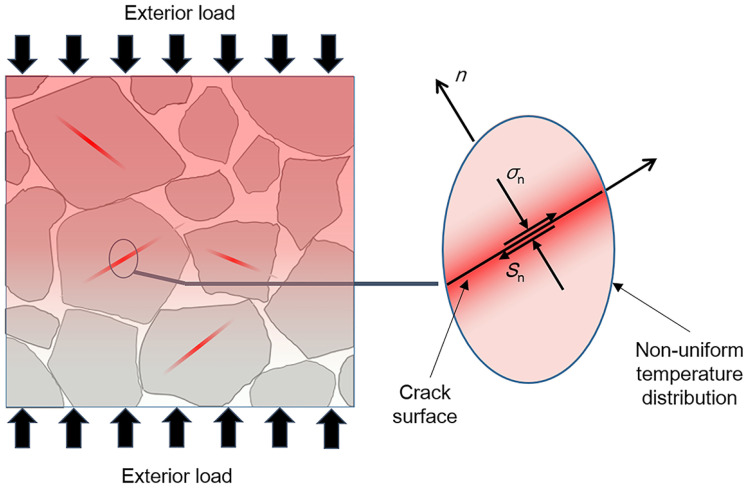
Heating process and non-uniform temperature distribution in PBX1314.

Along with damage accumulation, PBXs would experience bulk temperature rise due to adiabatic compression, viscous dissipation or other mechanisms when exposed to exterior stimuli. Meanwhile, interfacial friction of microcracks in PBXs leads to heat generation and accumulation near the microcracks. Heat accumulation in local regions results in non-uniform temperature distribution in PBXs. The heating process and non-uniform temperature distribution are illustrated in Figure 6. Hotspots’ formation or even initiation might occur due to the non-uniform temperature field. In this section, a non-shock initiation model of PBX1314 is presented to study the temperature evolution properties of PBX1314 related to different heating mechanisms. A non-uniform initiation criterion of PBX1314 under mild stimuli is developed. 

### 4.1. Bulk Temperature Increase in PBX1314

Bulk temperature increase in PBX1314 comes from different heating sources [19,20]. The energy balance equation is

(27) $${\dot{T}}_{\mathrm{bulk}}=\alpha T_{\mathrm{bulk}}{}_{,ii}-\gamma T_{\mathrm{bulk}}{\dot{\varepsilon }}_{jj}+\left(\xi /\rho C_{V}\right)\left(P_{ve}+P_{cr}\right)+\left(Q_{r}/C_{V}\right)Z\exp\left(-E/RT\right)$$

The *T*_bulk_ is the time rate of bulk temperature. On the right-hand side, the first term represents heat conduction. The letter *α* = *k*/(*ρC*_V_) is heat diffusion coeffecient. It is a function of thermal conductivity *k*, mass density *ρ*, and heat capacity at constant volume *C*_V_. The second term describes the heating results from adiabatic compression. The letter *γ* is the Gruneisen coefficient. The minus before this term denotes that compression leads to a temperature rise while expansion results in a temperature decrease. The third term is dissipation heating due to viscosity and crack damage. The *ξ* is a transition constant that converts viscous work power *P_ve_* and crack work power *P_cr_* to heating rate. The two powers are

(28) $$P_{ve}=\sum_{1}^{N}\frac{s_{ij}^{\left(n\right)}s_{ij}^{\left(n\right)}}{2G^{\left(n\right)}\tau^{\left(n\right)}}$$

(29) $$P_{cr}=\frac{1}{2G}[3{\left(\frac{c_{a}}{a}\right)}^{2}\frac{{\dot{c}}_{a}}{a}s_{ij}s_{ij}+{\left(\frac{c_{a}}{a}\right)}^{3}s_{ij}{\dot{s}}_{ij}]$$

The term (*Q*_r_/*C*_v_)*Z*exp(-*E*/*RT*) on the right-hand side of Equation (27) describes chemical reaction heating. The *Q*_r_ is reaction heat per unit mass of PBX from chemical decomposition, *Z* is a pre-exponential factor, *E* the activation energy, *R* the universal gas constant.

### 4.2. Energy Dissipation and Accumulation Near Microcracks

To study the non-uniform heating mechanism in PBX1314, the dominant crack in the dynamic damage model is employed and the non-uniform temperature distribution caused by the interfacial friction heating of the dominant crack is investigated. The heated region is assumed to be much thinner than the radius of the dominanat microcrack. Therefore, the heat conduction caused by the frictional heat flow could be regarded as one-dimensional and vertical to the crack surface, which is illustrated in Figure 6. The heat flux on the crack surface is

(30) $$\dot{q}=\mu \langle \sigma_{n}\rangle v_{s}$$

where *v*_s_ is the efficient sliding velocity of the crack surface and expressed as

(31) $$v_{s}=\left(3\beta /2\pi \right)[\langle {\dot{s}}_{n}+\mu {\dot{\sigma }}_{n}\rangle c_{a}+\langle s_{n}+\mu \sigma_{n}\rangle {\dot{c}}_{a}]$$

The two terms in the square bracket are sliding velocities results from a change in traction on the crack surface and growth of crack size. The static or sliding state of the microcrack determines that the friction parameter *μ* equals static friction coefficient *μ*_s_ or dynamic friction coefficient *μ*_d_. 

The heat conduction besides the crack surface could be simplified as one-dimensional and the direction is normal to the crack surface. The heat conduction equation is

(32) $$\rho C_{V}\dot{T}=kT_{xx}+\rho Q_{r}Z\exp\left(-E/RT\right)$$

where *T* is the temperature near the microcrack, *Q*_r_ is the chemical reaction heat per unit mass of PBX1314, *E* is the activation energy, *R* is the general gas constant. In this work, the initial temperature is set to be 300 K throughout PBX1314. The temperature of the zones which are far away from the microcrack surface is assumed to be *T*_bulk_. On the crack surface, the heat flux is

(33) $$-kT_{x}=\dot{q}$$

The evolution and distribution of temperature near the microcrack could be obtained by solving Equation (32).

### 4.3. Temperature Increase in PBX1314 under Dynamic Loads

The non-shock initiation model is converted into a VUMAT code by using FORTRAN and then implemented into ABAQUS. The dynamic damage model in Section 3 and the non-shock initiation model in this section together make up the dynamic damage and initiation model of PBX1314. The dynamic damage and initiation model is employed to calculate the temperature increase when PBX1314 is under uniaxial compression and at the strain rate of 2000 s^−1^. The initiation model parameters of PBX1314 is listed in Table 1. 

**Figure 7:**
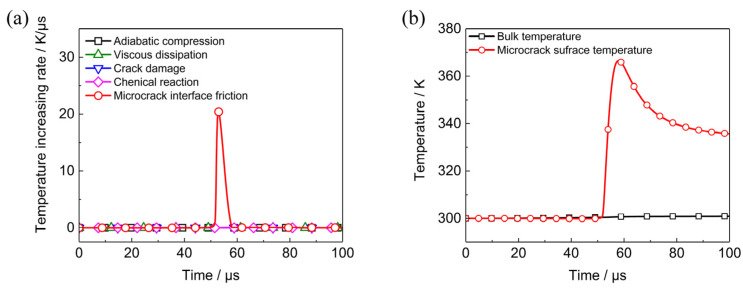
Temperature increasing rates due to different mechanisms and the resulting evolutions of representative element volume (RVE) bulk temperatures and microcrack surface temperature when PBX1314 is under dynamic uniaxial compression and at the strain rate of 2000 s^−1^. (**a**) Temperature increasing rates due to five different mechanisms, (**b**) RVE bulk temperature and microcrack surface temperature.

Initial temperature of PBX1314 is assumed to be 300 K. Heating rates results from five different mechanisms are plotted in Figure 7a. The peak value of microcrack interface friction heating rate is 20 K/μs, which is much larger than the other four heating rates. The heating flow corresponding to the peak heating rate is 2.2 × 10^7^ J·m^−2^·s^−1^. The evolution of the bulk temperature and microcrack surface temperature is plotted in Figure 7b. The bulk temperature increase is only 1 K, while the microcrack surface temperature is larger than 60 K. This is because the microcrack interface friction heating rate is three orders of magnitude larger than the bulk heating rates and the frictional heat could not flow into neighbouring regions instantly.

The calculation above reveals that the microcrack interface temperature increase is remarkable, while the bulk temperature rise is tiny. The difference suggests that the temperature distribution is not uniform. Microcrack surface temperature increase might lead to formation of hot spots or even initiation once the applied load is strengthened and the strain rate is enhanced. 

**Figure 8:**
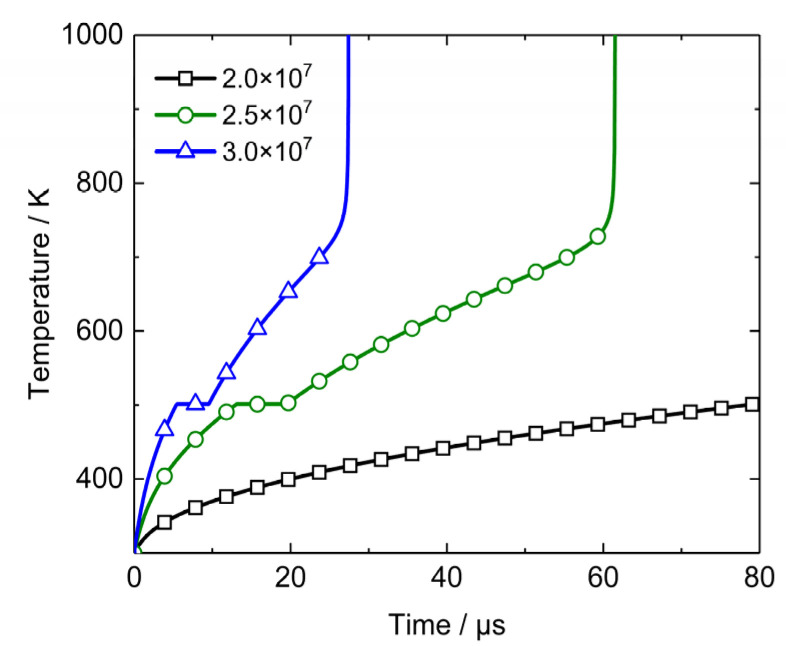
Temperature histories on a microcrack surface with different boundary heat flows.

To investigate the non-uniform heating property of PBX1314, a series of calculations are conducted. For simplicity, the microcrack size is set to be 30 μm and the heat flux in Equation (30) is assumed to be constant. The bulk temperature increase rate is ignored because it is much lower than the microcrack interfacial friction heating rate. Initial temperature of PBX1314 is 300 K. In view of the peak value of heating flow in Figure 7, the constant heat flux is set to be 2.0 × 10^7^, 2.5 × 10^7^, and 3.0 × 10^7^ J·m^−2^·s^−1^, respectively. The heat flux sustains 80 μs and could be considered as a rectangular wave. Evolutions and distributions of the microcrack surface temperature resulting from different constant heat flux rectangular waves are calculated. Microcrack surface temperature histories are plotted in Figure 8. When the heating flow is 2.0 × 10^7^ J·m^−2^·s^−1^, the temperature increase rate is low so that PBX1314 does not melt within 80 μs. As the heating flow increases to 2.5 × 10^7^ J·m^−2^·s^−1^, the melt of PBX1314 occurs in 13 μs. The continuing heating triggers a chemical reaction and results in a steep rise in the microcrack surface temperature. In the case of 3.0 × 10^7^ J·m^−2^·s^−1^, material melt and the steep rise of the temperature proceed in a shorter period.

**Figure 9:**
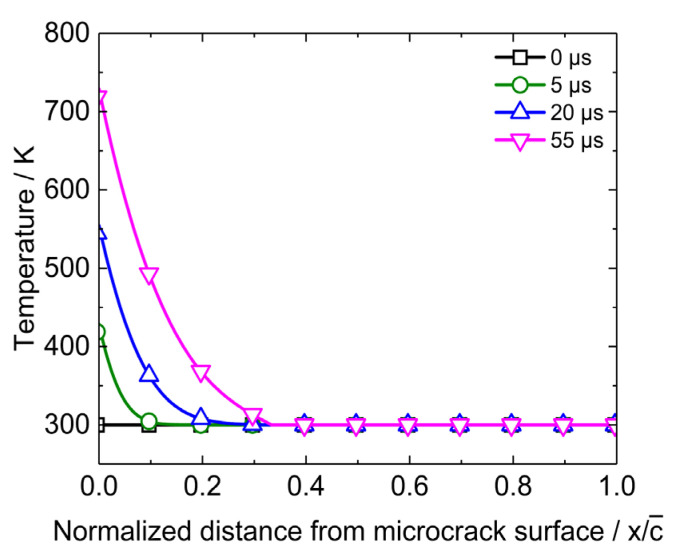
Distribution of temperature near the microcrack.

Temperature distribution near the microcrack when the heat flux is 2.5 × 10^7^ J·m^−2^·s^−1^ is shown in Figure 9. In view of symmetry, only the temperature on one side of the microcrack surface is plotted. The horizontal axis is the ratio of the distance to the microcrack surface to the micorcrack radius. Figure 9 indicates that the frictional heat accumulates near the microcrack surface and leads to temperature increase. The maximum of the temperature is on the microcrack surface. The temperature drops rapidly as the distance to the microcrack surface increases. It decreases to bulk temperature when the distance is 0.3*c_a_*. The temperature distribution in Figure 9 implies that the accumulation of frictional heat leads to a symmetric high temperature zone near the microcrack. The feature size of the zone is relatively small and the temperature in this zone is higher than the bulk temperature. 

The calculation and analysis above suggest that microcrack friction is a heterogeneous heating mechanism which results in non-uniform temperature field. The microcrack surface temperature might exceed 700 K which causes initiation of PBX1314, although the bulk temperature remains in a low level. Therefore, the initiation criterion could be developed based on the non-uniform heating. 

### 4.4. Non-Uniform Initiation Criterion

Heat accumulation and temperature rise near the microcrack surface would lead to the chemical reaction of PBX1314. This threatens the safety of PBX1314 in military weapons. It is necessary to develop a criterion to determine whether non-shock initiation occurs when PBX1314 is exposed to mild stimuli. The heat and temperature near the microcrack surface are both variables in mesoscale. However, it is difficult and inconvenient to assess the safety of PBX in engineering by using mesoscale variables. Macroscale variables are convenient instead. In Section 3, the macroscale stress state of PBX1314 could be calculated by using the proposed dynamic damage model. The frictional heat flux on the microcrack surface could be calculated once the macroscale stress state is known. Then, the feature of the high temperature zone could be obtained. We would seek a non-shock initiation criterion. In the criterion, the local non-uniform temperature field near microcrack surface in mesoscale is taken into full consideration. In addition, the final form of the criterion is expressed by the macroscale state variables and macroscale material parameters of PBX1314 so that it is convenient for engineering applications.

**Figure 10:**
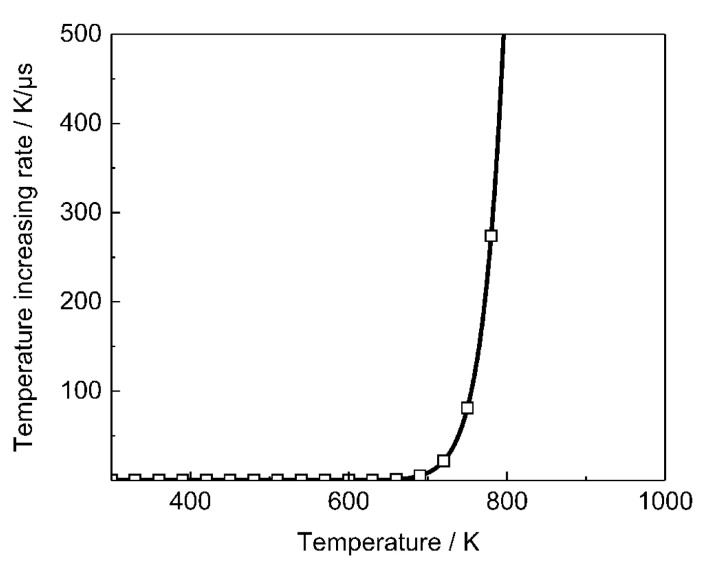
Temperature increasing rate calculated by Arrhenius reaction model.

The heating rate at different temperatures calculated by the Arrhenius chemical reaction model is given in Figure 10. The steep rise at 700 K indicates that initiation is activated. Therefore, it is reasonable to consider the initiation temperature as 700 K. The initiation time *t*_initiation_ is defined as the time that the microcrack surface temperature increases from bulk temperature to initiation temperature under the condition of a constant heat flux rectangular wave. 

**Figure 11:**
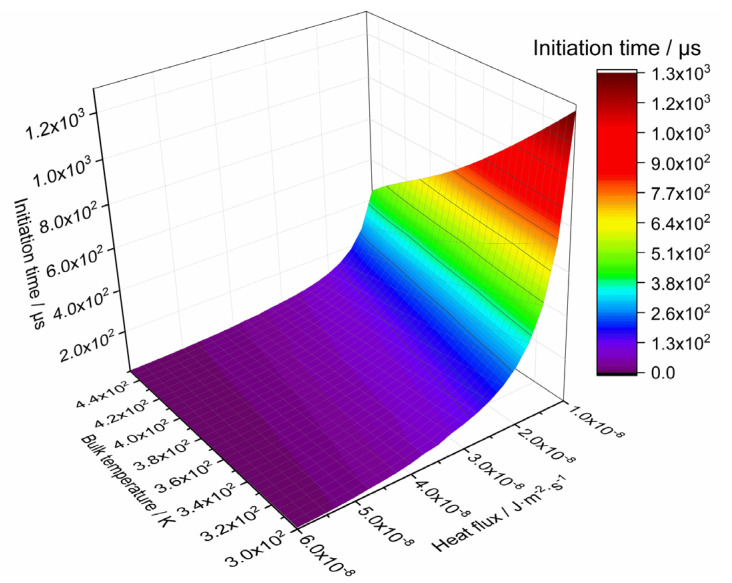
Initiation times under the condition of different bulk temperatures and microcrack surface heat flows.

According to the definition of initiation temperature above, the initiation times with different bulk temperatures and under different constant heat flux rectangular waves are calculated. A curved surface is obtained by the fitting of the data of initiation times and displayed in Figure 11. For a given combination of bulk temperature and constant heat flux rectangular wave, the initiation of PBX1314 would occur once *t*_load_ exceeds *t*_initiation_. The fitting of the curved surface in Figure 11 gives the relationship

(34) $$t_{in}=A_{in}\left(T_{b}\right)\cdot {\left\{\mu_{d}\sigma_{n}\frac{3\beta }{2\pi }\left[\langle {\dot{s}}_{n}+\mu {\dot{\sigma }}_{n}\rangle c_{a}+\langle s_{n}+\mu \sigma_{n}\rangle {\dot{c}}_{a}\right]\right\}}^{B_{in}\left(T_{b}\right)}$$

where

(35) $$A_{in}\left(T_{b}\right)=P_{1}\cdot \exp\left(P_{2}\cdot T_{b}\right)+P_{3}\cdot \exp\left(P_{4}\cdot T_{b}\right)$$

(36) $$B_{in}\left(T_{b}\right)=P_{5}\cdot \exp\left(P_{6}\cdot T_{b}\right)+P_{7}\cdot \exp\left(P_{8}\cdot T_{b}\right)$$

The coefficients in the three equations above are listed in Table 2. The material in different positions of the PBX1314 in the earth-penetrating-projectile during penetration would experience stimuli in different durations. The bulk temperature and stress state are variables in macroscale and could be calculated by using the dynamic damage and initiation model and the initiation time *t*_in_ could be obtained from Equation (34). The initiation of PBX1314 would occur once the duration of stimuli exceeds *t*_in_

(37) $$\Delta t_{load}>t_{in}$$

## 5. Damage and Initiation of PBX1314 during Penetration

In this section, the dynamic damage and initiation model of PBX1314 is employed to investigate the mechanical and thermal responses of PBX1314 during penetration. The effects of microcracks and binder on the damage and initiation of PBX1314 are analyzed.

### 5.1. Finite Element Model

**Figure 12:**
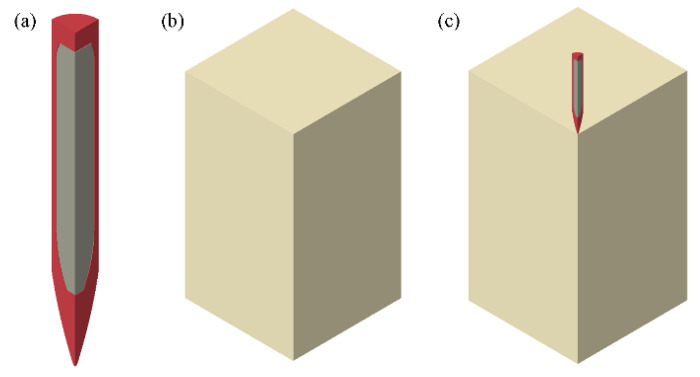
Finite element model of the penetration of an earth penetrating missile (EPM) with PBX1314 charge into a concrete target. (**a**) a missile (the red part) with PBX (the grey part), (**b**) a concrete target, (**c**) assembly of the missile and the target.

The finite element model of penetration is shown in Figure 12. In consideration of symmetry, only a quarter of the geometry model is created and calculated. Symmetric boundary conditions are applied to the symmetry planes. The projectile impacts the target with different initial velocities. PBX1314 is bonded with the projectile by a layer, which performs with weak strength. Cohesive elements with weak strength are created at the interface of the PBX1314 and the projectile to model this layer. 

The mechanical parameters of PBX1314 in Table 1 are used in the finite element model. An elastic–plastic model is used to characterize the mechanical behaviour of the steel projectile. The density is 8020 kg/m^3^, the Young’ s modulus 200 GPa, the Poisson ratio 0.3, the yield stress 1.72 GPa, and the hardening modulus 1.72 GPa. The bonding layer is modelled by a material with 1100 kg/m^3^ density, 250 MPa Young modulus, and 20 J/m^2^ fracture energy. The HJC model [31] is employed to characterize the dynamic response of the concrete target. It is implemented into ABAQUS by using a user material subroutine. As the concrete in our experiments has similar properties to that of Holmquist. et al. [31], the constitutive parameters [31] are used in this finite element model.

### 5.2. Results of the Model that the Friction between Projectile Shell and PBX1314 Is Not Considered

#### 5.2.1. Deformation of PBX1314 Main Explosive during Penetration

**Figure 13:**
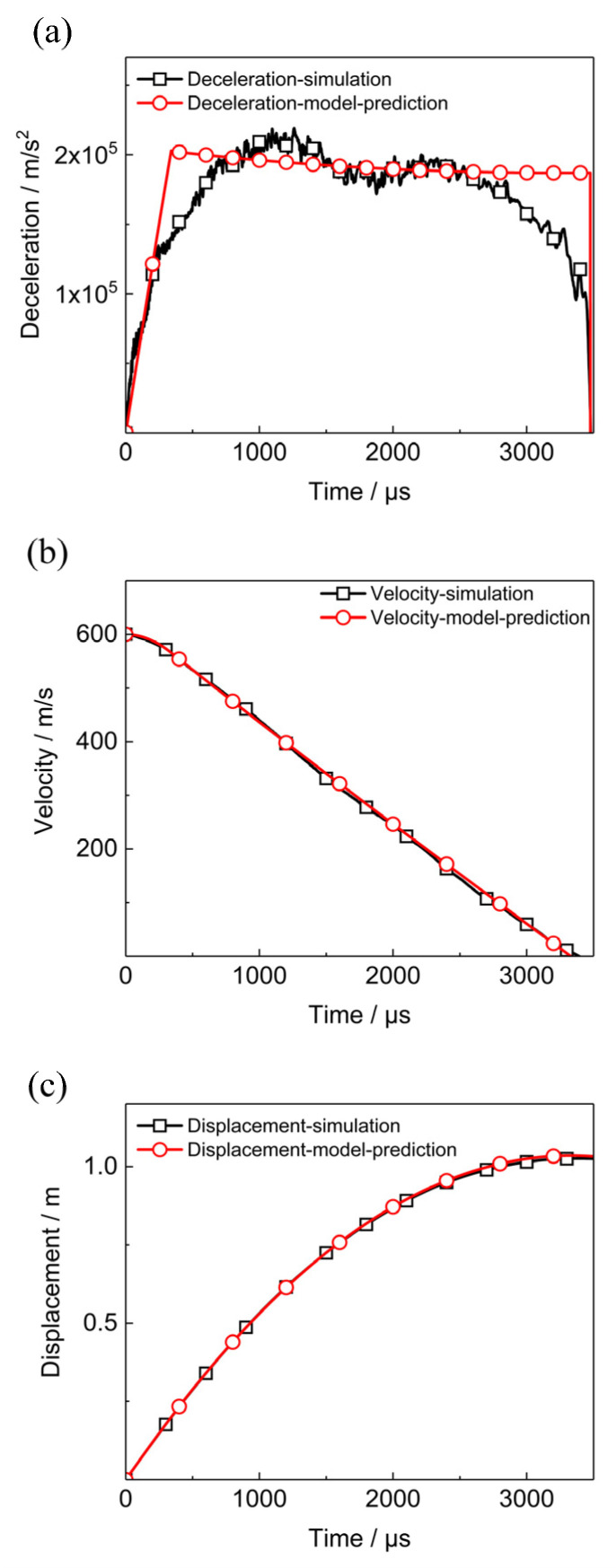
Comparisons of the calculated deceleration, velocity, and displacement of the EPM in the simulation and the corresponding variables predicted by using the cavity expansion theory. (**a**) Decceleration, (**b**) velocity, (**c**) displacement.

The simulated deceleration, velocity and displacement of the projectile in the case of the initial impact velocity is 602 m/s are plotted in Figure 13. Predictions of the deceleration, velocity, and displacement based on the cavity expansion theory [32] are also shown in Figure 13. These agreements between the predictions and the simulated results verify the creditability of the simulation results of the finite element model.

**Figure 14:**
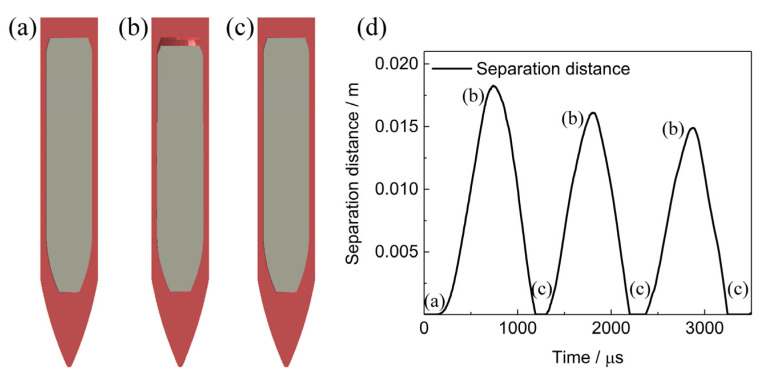
Deformation of the PBX1314 in the projectile. (**a**) The initial state. (**b**) Separation in the tail. (**c**) Impact in the tail part. (**d**) Separation distance of the tail surfaces of PBX1314 and the projectile.

Deformation of PBX1314 is analyzed in the following. PBX1314 is initially bonded with the projectile in Figure 14a. During penetration, compressive stress waves transform into tensile reflective stress waves in the tail of the projectile. The tensile waves propagate to the interface between the projectile and the tail of PBX1314. Debonding occurs on this interface due to the weak strength of the bonding layer. Inertia force and low modulus of PBX1314 cause forward displacement of the tail part and compression of the head part of PBX1314. The debonding and compression are illustrated in Figure 14b. Along with the compression, input energy to PBX1314 is partially stored as elastic energy and partially dissipated due to adiabatic compression, viscous effect and crack damage. With the proceeding of penetration, the deceleration decreases and so does the inertia force of PBX1314. In contrast, the stored energy in the head of PBX1314 accumulates to a high level. The effect of elastic energy exceeds that of the inertia force in a certain moment and the rebound of PBX1314 occurs. The tail of PBX1314 impacts the projectile. The rebound and impact are shown in Figure 14c. PBX1314 undergoes three compression–rebound cycles. Variation in the separation distance between the tail of PBX1314 and projectile is plotted in Figure 14d.

#### 5.2.2. Damage Evolution and Heat Localization in PBX1314

**Figure 15:**
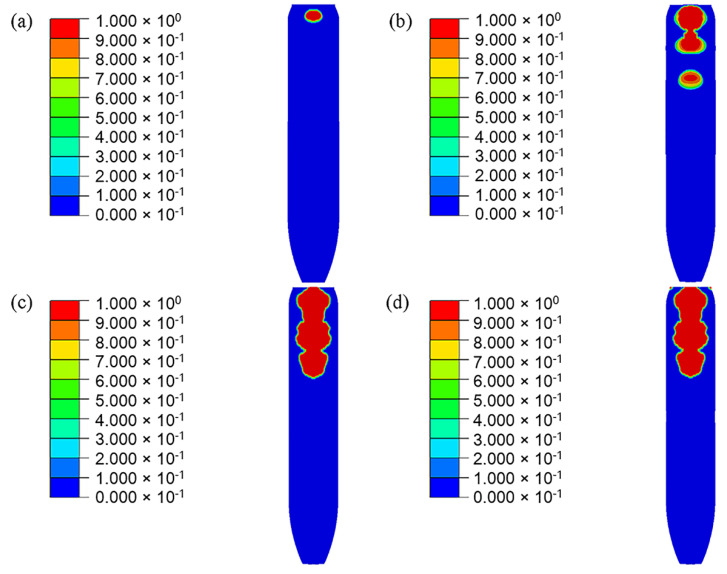
Evolution of the microcrack damage of the PBX1314 main explosive. (**a**) 500 μs. (**b**) 1200 μs. (**c**) 2200 μs. (**d**) 3400 μs.

**Figure 16:**
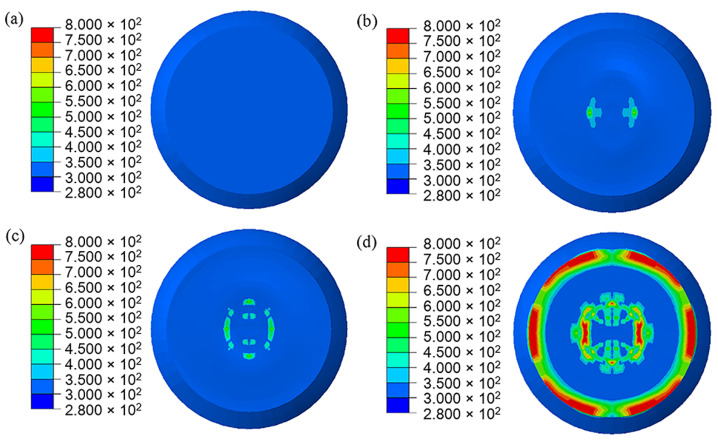
Evolution of the microcrack surface temperature of the PBX1314 main explosive. (**a**) 500 μs. (**b**) 1300 μs. (**c**) 2300 μs. (**d**) 3300 μs.

In the simulation results, the head part of PBX1314 is always compressed while the tail part undergoes three impacts with the projectile. This results in a non-uniform distribution of damage in PBX1314 in Figure 15. Severe damage ranges from the tail to the middle region, while the head is rarely damaged. This agrees with the experimental results in Section 2. Figure 15 reveals that PBX1314 damages mainly in the first two compression–rebound cycles in Figure 14d. This is due to the severe impacts in the first two cycles. Microcrack surface temperature distribution on the tail surface of PBX1314 is shown in Figure 16. It reveals that the first and second impacts lead to heat generation and localization on the tail surface of PBX1314. Weak chemical reactions are triggered near some microcrack surfaces. The third impact results in initiation near these sites.

**Figure 17:**
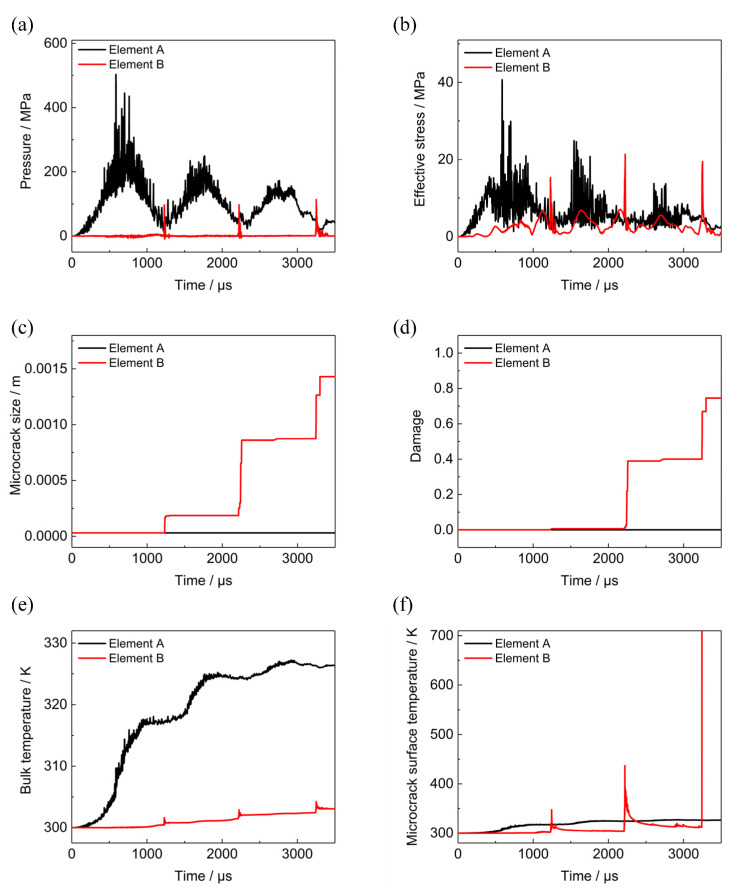
History curves of several variables of Element A and B. (**a**) Pressure. (**b**) Effective stress. (**c**) Microcrack size. (**d**) Damage. (**e**) Bulk temperature. (**f**) Microcrack surface temperature.

To gain more knowledge of damage evolution and heat localization, two elements are selected in the head and tail of PBX1314 and labelled as A and B, respectively. The historical information of stresses, microcrack growth, damage, and temperatures of the two elements are plotted in Figure 17.

As the head region of PBX1314 is always compressed in the three compression–rebound cycles, the hydrostatic stress of Element A is positive throughout. The peaks in the pressure and effective stress curves of Element A in Figure 17a,b correspond to the three compressions. The effective stress is in such a low level that the shear stress on the dominant microcrack surface does not exceed the static friction. All microcracks in Element A are in a friction-locked state and do not grow or slide. Therefore the crack size and damage of Element A in Figure 17c,d remain their initial values. In Element B, there are also three peaks in pressure and effective stress curves. These peaks relate to the three impacts on the tail end of PBX1314. As the peak pressure of Element B is not at a high level, the shear stress on the dominant microcrack surface of Element B overcomes friction and the dominant microcrack becomes unstable. Crack growth and damage accumulation occur in Element B. They are shown in Figure 17c,d.

**Figure 18:**
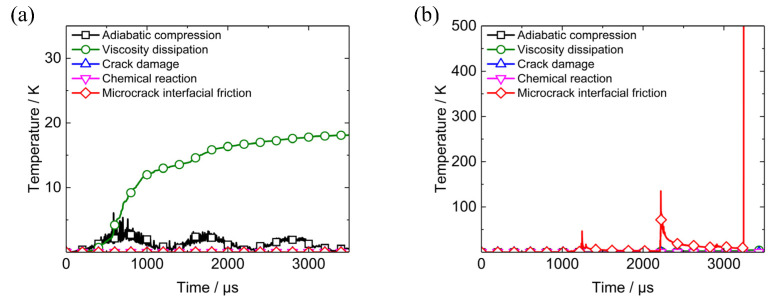
Temperature increases in Element A and B due to different heating mechanisms. (**a**) Element A, (**b**) Element B.

Bulk temperatures and microcrack surface temperatures of the two elements are plotted in Figure 17e,f. The temperature rises in the two elements due to different heating mechanisms, are shown in Figure 18. The curves in Figure 18a reveals that viscous dissipation contributes greatly to the bulk temperature rise in Element A and no microcrack surface heating occurs because of the friction-locked state of the dominant microcrack. Therefore, the microcrack surface temperature equals the bulk temperature in Element A all the while. This indicates that the temperature distribution in Element A is uniform. The temperature is much lower than 700 K and initiation does not occur. 

In Element B, the bulk temperature in Figure 17e is relatively low because the temperature rise due to bulk heating mechanisms in Figure 18b remains in a low level throughout the penetration. However, the three impacts between PBX1314 and projectile activate microcrack growth, sliding, and friction in tail end of PBX1314. The friction leads to three peaks in the microcrack interfacial friction heating curve in Figure 18b and in the microcrack surface temperature curve in Figure 17f. The first two peaks of the microcrack surface temperature curve are, respectively, 350 and 450 K, while the third exceeds 700K, which might trigger initiation. 

During the first two impacts, the frictional heat flows on the microcrack surface are 4.0 × 10^6^ and 4.0 × 10^7^ J·m^−2^·s^−1^. The initiation time calculated by Equation (34) are, respectively, 2687.49 and 38.55 μs. The non-shock initiation criterion in Equation (37) is not satisfied because the durations of the first two impacts in the simulation results are only 2 and 3 μs. Along with the third impact, the microcrack radius increases to five times its initial size. Crack sliding velocity is enhanced to a high level and the heat flow exceeds 4.5 × 10^9^ J·m^−2^·s^−1^. The initiation time obtained by Equation (34) is 6.72 × 10^−3^ μs. The criterion in Equation (37) is satisfied and initiation occurs in Element B.

#### 5.2.3. Reaction Process after Initiation of PBX1314

In the simulation results, initiation of Element B occurs along with the third impact of PBX1314 with the tail of the projectile. Mutual promotion of chemical reaction and temperature rise would result in the complete reaction of Element B. However, the ignition, deflagration or detonation of PBX1314 could not be characterized accurately by the Arrhenius reaction model in the dynamic damage and initiation model of PBX1314. Besides, the third impact is transient. Whether it leads to complete reaction of Element B remains to be researched. In our previous work [25], the ignition and growth model (IGM) of PBX1314 has been constructed. This model is employed in this section to study the reaction process of Element B. 

**Figure 19:**
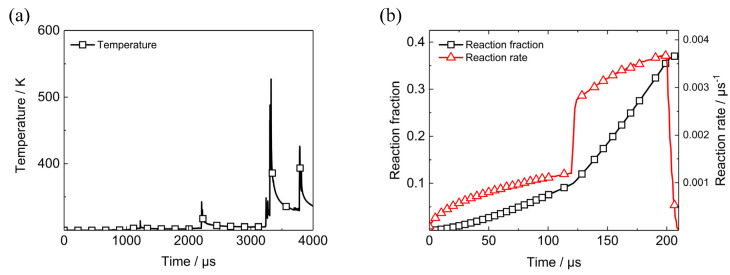
Temperature and reaction process in Element B. (**a**) Temperature history on the microcrack surface, (**b**) reaction fraction and reaction rate.

The curve in Figure 10 indicates that chemical reaction in PBX1314 could be ignored if the temperature is lower than 700 K. Therefore, the chemical reaction term is removed from the dynamic damage and initiation model and the microcrack surface temperature of Element B during penetration is re-calculated. The maximum of the temperature in Figure 19a is 550 K at 3325 μs. In the IGM of PBX1314, the equation of state (EOS) of unreacted material is

(38) $$p=A\exp\left(-R_{1}V_{s}\right)+B\exp\left(-R_{2}V_{s}\right)+\omega C_{V}T/V_{s}$$

in which *V*_s_ is the relative volume of PBX1314. In the simulation results, the volumetric strain of Element B is 0.01 at the moment of the third impact. Then, *V*_s_ is calculated to be 0.99. For simplicity, the maximums of pressure and microcrack surface temperature of Element B at the moment of the third impact are used to calculate the reaction process of Element B by the IGM. The duration is 200 μs. The obtained reaction rate and reaction fraction are given in Figure 19b. Ignition occurs in the first 125 μs, but the reaction rate is always below 0.001 μs^−1^. With the proceeding reaction, the reaction fraction exceeds 0.1 and fast reaction is triggered. The reaction rate increases to 0.003 μs^−1^ and the final reaction fraction is 0.4, which indicates incomplete reaction of Element B. This coincides with the initiation and quench in the penetration experiments in Section 2.

The simulated damage distribution, non-shock initiation, and incomplete reaction of PBX1314 show good agreement with the penetration experiment results. This demonstrates that the combination of the dynamic damage and initiation model and IGM performs well in predicting the mechanical, thermal, and chemical reaction responses of PBX1314 during penetration. 

### 5.3. Effects of Friction between Projectile Shell and PBX1314

**Figure 20:**
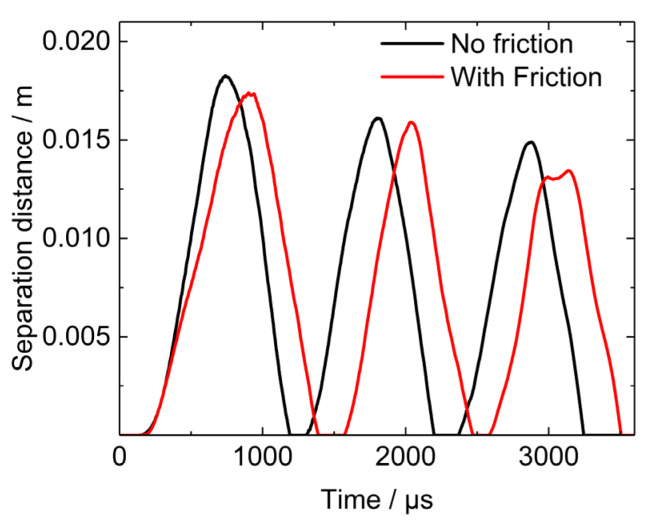
Evolution of separation distance between the tail of PBX1314 and projectile (The curve ‘No friction’ has been displayed in Figure 14b).

**Figure 21:**
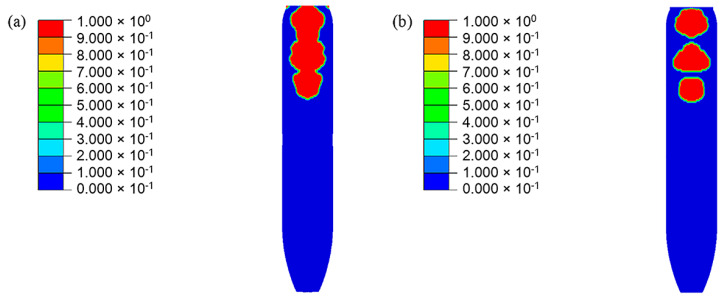
Damage distribution in PBX1314. (**a**) No friction between the projectile and PBX1314 (It corresponds to the Figure 15d.), (**b**) Friction exists between the projectile and PBX1314.

The role of friction is important, especially the friction between the microcrack surfaces. In this work, the friction was taken into consideration in a newly calculated model. The friction coefficient is assumed to be 0.1, according to the reference [33]. In the new calculation results, the evolution of the separation distance between the tail of PBX1314 and the projectile is shown in Figure 20. This indicates that the friction between the projectile shell and PBX1314 could mildly weaken and postpone the deformation of compression–rebound cycles of PBX1314. The damage distribution in the end of the penetration is displayed in Figure 21. The distribution of the damaged zone is similar to that when the shell-PBX1314 friction is not considered. As the friction weakens the compression and rebound of PBX1314, the impact of the tail of PBX1314 on the projectile is weakened, and the damaged zone is little bit smaller. 

**Figure 22:**
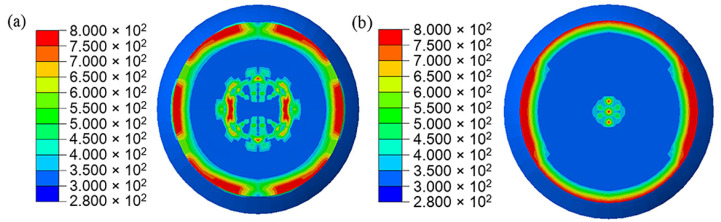
Initiation zone in PBX1314. (**a**) No friction between the projectile and PBX1314 (It corresponds to the Figure 16d in the manuscript), (**b**) Friction exists between the projectile and PBX1314.

**Figure 23:**
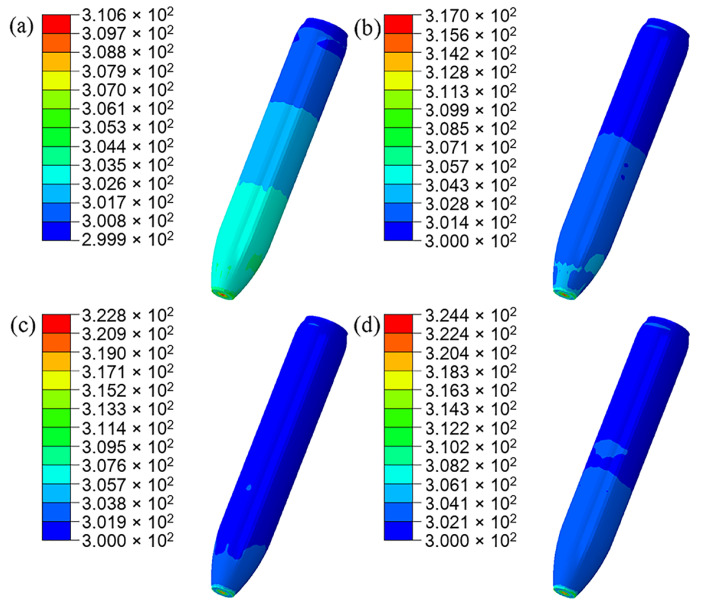
The evolution of the microcrack surface temperature on the lateral surface the PBX1314. (**a**) 800 μs, (**b**) 1600 μs, (**c**) 2400 μs, (**d**) 3300 μs.

Non-shock initiation occurs on the tail surface of PBX1314. The initiated zone is illustrated in Figure 22. As the impact between the tail of PBX1314 and projectile is weakened due to the shell-PBX1314 friction, heat generation and localization near the microcracks is weakened. Therefore, the initiation zone in Figure 22b is smaller than that in Figure 22a. The temperature distribution on the lateral surface of PBX1314 is shown in Figure 23. The shell-PBX1314 friction results in a temperature rise on the lateral surface of PBX1314. However, the temperature is far below the local initiation temperature of PBX1314 (700 K).

The calculation and analysis above suggest that the friction on the interface of the projectile shell and PBX1314 weakens the deformation, mechanical damage evolution, and non-shock initiation of PBX1314 during penetration. However, in general, the effect is limited. The simulation including the shell-PBX1314 friction also predicts the penetration experiment phenomenon.

## 6. Discussion

The complexities of material composition and loads make it difficult to investigate damage and non-shock initiation of PBX. In this paper, penetration experiments are conducted to study the mechanical and thermal responses of PBX1314. A dynamic damage and initiation model and a non-shock initiation criterion are proposed. The model and criterion successfully predict the mechanical damage and non-shock initiation of PBX1314 in experiments. 

In dynamic impact experiments [26], microcrack growth causes damage and macroscopic fracture in PBX1314. Similar processes occur in penetration experiments and simulations in this work. It suggests that the unstable growth of microcracks cause mechanical damage in PBX1314 during penetration. In the analysis of impact initiation experiments, Dienes [34] suggests crack friction to be the most likely mechanism that results in hotspots’ formation and initiation, since the heating rate of crack friction is two orders of magnitude larger than the heating rates of other mechanisms. This idea has been adopted [4,5,19] as a basic assumption of different investigations on impact initiation. Calculations based on microcrack theories provide evidences to this assumption and viable explanations to a series of experiments. In this paper, the temperature increases due to adiabatic compression, inelastic work, and chemical reactions below 600 K are not high enough to initiate PBX1314. In contrast, the microcrack friction heating rate is as high as 6000 K/μs. It plays significant roles in the temperature rise and the non-shock initiation. The non-shock initiation criterion based on macroscale variables is, in essence, an energy criterion because the accumulation of heating flow over time is the energy per unit area. This suggests that there exists an energy threshold beyond which the initiation of PBX1314 is activated. These resemble the *pτ*^2^ criterion and the dual ignition criterion [35] although they are proposed on the basis of different heating mechanisms. 

## 7. Conclusions

Dynamic mechanical damage and non-shock initiation of PBX1314 during penetration are experimentally studied in this paper. PBX1314 damages from the tail end to the middle. Non-shock initiation occurs on the tail surface of PBX1314 but the material in the initiated regions is not completely reacted. The mechanical, thermal, and chemical responses of PBX1314 in the penetration experiments are successfully predicted by using the proposed dynamic damage and initiation model and non-shock initiation criterion. The predicted result reveals that PBX1314 undergoes compression and rebound repeatedly during penetration. The tail of PBX1314 impacts the projectile several times, which leads to severe damage in the tail and middle part of PBX1314. Microcrack Interfacial friction plays significant roles in the formation of non-uniform temperature distribution in PBX1314. Along with the third impact, the initiation criterion is satisfied due to localization of the frictional heat near microcrack surfaces and non-shock initiation occurs in the tail of PBX1314. The material in the initiated region reacts incompletely because the input energy to Element B in the third impact is not enough to support a complete reaction. The friction between the projectile shell and PBX1314 weakens the deformation, mechanical damage evolution and non-shock initiation of PBX1314 during penetration. However, the effect is limited. 

## Figures and Tables

**Table 1:** Material parameters for PBX1314.

Parameter	Value	Parameter	Value
*G*^(1)^ (MPa)	472	*v*_max_ (m/s)	300
*G*^(2)^ (MPa)	115.94	*c*_0_ (m)	0.00003
*G*^(3)^ (MPa)	83.56	*γ*_0_ (J·m^−2^)	50
*G*^(4)^ (MPa)	4.27	*ρ* (kg·m^−3^)	1690
*G*^(5)^ (MPa)	35	*μ*_s_	0.5
*τ*^(1)^(μs)	0.75	*μ*_d_	0.2
*τ*^(2)^ (μs)	7.5	*C*_V_ (J·Kg^−1^·K^−1^)	970
*τ*^(3)^ (μs)	75	*k* (J·m^−1^·s^−1^·K^−1^)	0.292
*τ*^(4)^ (μs)	750		
*τ*^(5)^ (μs)	∞	*T_m_* (K)	501.9
*ν*	0.49	*Q_r_* (J·Kg^−1^)	2.09 × 10^6^
*m*	10	*E* (J·mol^−1^)	2.37 × 10^5^
*a* (m)	0.002		

**Table 2:** Coefficients of the initiation time surface.

Coefficient	Value	Coefficient	Value
*P*_1_	6.834 × 10^−12^	*P*_5_	9.711 × 10^−6^
*P*_2_	1.422 × 10^−3^	*P*_6_	2.010 × 10^−2^
*P*_3_	2.849 × 10^−19^	*P*_7_	−1.931
*P*_4_	3.550 × 10^−2^	*P*_8_	−1.639 × 10^−4^
